# Nutritional control of IL-23/Th17-mediated autoimmune disease through HO-1/STAT3 activation

**DOI:** 10.1038/srep44482

**Published:** 2017-03-14

**Authors:** Jürgen Brück, Julia Holstein, Ivana Glocova, Ursula Seidel, Julia Geisel, Toshio Kanno, Jin Kumagai, Naoko Mato, Stephan Sudowe, Katja Widmaier, Tobias Sinnberg, Amir S. Yazdi, Franziska C. Eberle, Kiyoshi Hirahara, Toshinori Nakayama, Martin Röcken, Kamran Ghoreschi

**Affiliations:** 1 Department of Dermatology, University Medical Center of the Eberhard Karls University Tübingen, 72076 Tübingen, Germany; 2 Department of Immunology, Graduate School of Medicine, Chiba University, Chiba 260-8670, Japan; 3 Department of Dermatology, University Medical Center of the Johannes Gutenberg University Mainz, 55101 Mainz, Germany

## Abstract

The nutritional curcumin (CUR) is beneficial in cell-mediated autoimmune diseases. The molecular mechanisms underlying this food-mediated silencing of inflammatory immune responses are poorly understood. By investigating antigen-specific immune responses we found that dietary CUR impairs the differentiation of Th1/Th17 cells *in vivo* during encephalomyelitis and instead promoted Th2 cells. In contrast, feeding CUR had no inhibitory effect on ovalbumin-induced airway inflammation. Mechanistically, we found that CUR induces an anti-inflammatory phenotype in dendritic cells (DC) with enhanced STAT3 phosphorylation and suppressed expression of *Il12b* and *Il23a*. On the molecular level CUR readily induced NRF2-sensitive heme oxygenase 1 (HO-1) mRNA and protein in LPS-activated DC. HO-1 enhanced STAT3 phosphorylation, which enriched to *Il12b* and *Il23a* loci and negatively regulated their transcription. These findings demonstrate the underlying mechanism through which a nutritional can interfere with the immune response. CUR silences IL-23/Th17-mediated pathology by enhancing HO-1/STAT3 interaction in DC.

Silencing inflammatory pathways by nutritionals may have sustainable impact on treatment strategies for chronic immune-mediated diseases. Despite the direct role of nutritionals as allergens or inducers of oral tolerance, increasing data suggests that dietary factors or their metabolites can affect the nature of T cell-mediated immune responses^1^. This has been intensively studied for dietary fibre metabolites like short-chain fatty acids in experimental models of colitis^2^ or allergic airway inflammation^3^. Another group of nutritional factors modifying the course of chronic inflammatory disorders consists of phytochemicals. Some phytochemicals seem to be beneficial in the treatment of cancer but also in certain settings of chronic inflammation. Among the well characterized phytochemicals are sulforaphane, resveratrol and curcumin. The isothiocyanate sulforaphane that naturally occurs in cruciferous vegetables has been reported to protect from cancer and from inflammatory autoimmune disease^4^,^5^. Other examples of nutritional compounds with anti-tumoral and anti-inflammatory activities are the natural phenol resveratrol^6^,^7^, and the polyphenol diferuloylmethane (curcumin; CUR). This component of turmeric isolated from the rhizome of *Curcuma longa* has been reported to have anti-tumor properties and to dampen inflammatory conditions^8^. As a nutritional product, CUR has been tested in several preclinical settings of T cell-dependent organ-specific inflammatory diseases including arthritis^9^, colitis^10^, diabetes^11^ or graft-versus-host disease^12^. Similarly, some studies reported a beneficial effect of CUR treatment in rodent models of multiple sclerosis^13^,^14^,^15^,^16^.

Different mechanisms have been proposed to explain the beneficial effects of CUR. While its anti-tumoral effects seem to be mediated by the regulation of tumor suppressor genes, cellular apoptosis and oxidative stress, its anti-inflammatory mechanisms are not fully understood. CUR has been suggested to affect the phenotype of immune cells like T cells and dendritic cells (DC) and to interfere with different signaling pathways like NF-κB, NRF2 and JAK/STAT^10^,^13^,^17^,^18^,^19^. The effects of CUR on DC and DC-associated signaling pathways are of special interest since DC are the most relevant cell type for initiating inflammatory autoimmune responses and for priming T cells. Yet, the exact mechanism by which CUR improves T cell-mediated autoimmune disease remains enigmatic.

Here we aimed to unravel the molecular mechanisms, by which CUR affects inflammatory T cell-mediated immune responses. Although CUR is part of the daily dietary intake, most investigators have used an artificial route of administration.

Therefore, we first show that the nutritional compound CUR in food pellets administrated orally protects from experimental encephalomyelitis in a murine model of multiple sclerosis. The protective effect of CUR was associated with a targeted suppression of the encephalomyelitis-inducing Th17 response *in vivo*. In contrast, CUR did not suppress airway inflammation induced by an *in vivo* Th2 response. To understand the mechanism responsible for the effects of CUR on Th17-mediated inflammation, we focused on the cellular and molecular events in T cells and antigen-presenting cells (APC). Non-toxic doses of CUR did not directly affect T cell cytokine expression, but instead strongly affected the phenotype of DC, as CUR preferentially enhanced phosphorylation of STAT3 in LPS-stimulated DC. Activated STAT3 binds to the promoter loci of *Il23a* and *Il12b* and negatively regulates their transcription. Interestingly, the activation of STAT3 by CUR in DC is mediated through heme oxygenase 1 (HO-1), a stress-response protein readily induced by CUR *in vitro* and *in vivo*.

## Results

### Feeding CUR protects from Th17 cell-mediated encephalomyelitis

**Figure 1:**
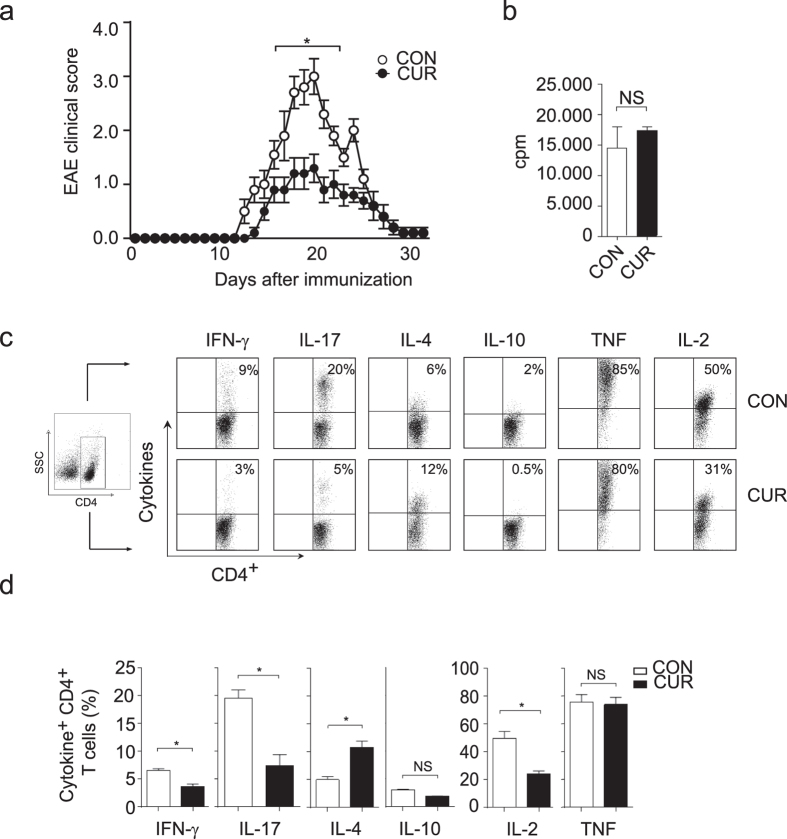
CUR protects mice from severe encephalomyelitis and inhibits IL-17 and IFN-γ production *in vivo*. (**a**) Mice were fed with 2% CUR or control diet (CON), immunized for EAE and followed for disease symptoms. The results show mean EAE score ± SEM from 2 independent experiments with n = 16 mice (*P < 0.05 day 16 to 22; Wilcoxon test). (**b**) Mice were fed with CUR as in (**a**) and immunized for EAE. On day 7 after immunization, CD4^+^ T cells were purified from draining lymph nodes and co-cultured with DC and PLP peptide. T cell proliferation was assessed by [^3^H] thymidine incorporation. NS: not significant, Mann-Whitney test. (**c,d**) Mice were treated as in (**a**). On day seven after immunization draining lymph nodes were isolated to analyze intracellular cytokine production in PMA/ionomycin-stimulated CD4^+^ T cells. First, a FSC/SSC plot was made and all lymph node cells were gated. The CD4^+^ T cell population was gated by a SSC/CD4 (left panel) plot and then analyzed for CD4^+^ T cell-specific cytokine expression. Dot plots from single mice are depicted in (**c**), pooled data from control mice (CON; n = 5) and CUR-treated mice (CUR; n = 5) are shown in (**d**). Bars represent the mean ± SEM (*P < 0.05, Mann-Whitney test).

The nutritional CUR has been reported to improve the outcome of experimental autoimmune encephalomyelitis (EAE) in rodents^13^,^14^,^15^,^16^ by using artificial and parenteral routes of administration. CUR was either dissolved in DMSO and injected intraperitoneally^13^,^16^ or encapsulated and delivered intranasally^15^. We asked if CUR also protects mice from EAE when given orally, the natural route of administration of CUR and other food additives like sulforaphane^5^ or fumarates^20^. Therefore mice were fed with 2% CUR-containing food pellets or received a control diet (CON) and were then immunized for the induction of EAE. The average daily consumption of CUR was 80.5 mg (Supplementary Fig. S1). Mice on CON diet immunized with PLP_139-151_ peptide developed a severe encephalomyelitis with clinical symptoms starting on day 12 after immunization and an average EAE score of 3.0 ± 0.31 SEM (Fig. 1a). In contrast, mice on oral CUR diet developed only mild symptoms of encephalomyelitis (average EAE score 1.3 ± 0.25 SEM). Accordingly, analysis of the central nervous system tissue of immunized mice on CUR diet showed less infiltration with mononuclear cells compared to mice on CON diet (Supplementary Fig. S2). Thus, the oral administration of this nutritional also protected from severe encephalomyelitis *in vivo*. To explain the clinical improvement of EAE by oral CUR administration, we first analyzed the influence of this dietary phytochemical on the activation of autoantigen-specific T cells. CUR diet had no significant impact on the proliferation of *in vivo* primed PLP_139-151_ peptide-specific CD4^+^ T cells (Fig. 1b). We therefore analyzed the cytokine phenotype of the CD4^+^ T cell responses in draining lymph nodes on day 7 after immunization with PLP_139-151_ peptide. Intracellular cytokine analysis of CD4^+^ T cells revealed a significant decrease of the pro-inflammatory cytokines IL-17, IFN-γ and IL-2 in mice receiving CUR diet (Fig. 1c,d), and an increase of the Th2 cytokine IL-4. T cell-derived IL-10 and TNF production remained unaffected. Thus, oral CUR administration impaired the development of Th17 and Th1 responses *in vivo*, while it promoted antigen-specific Th2 cells.

### Oral CUR does not improve Th2-mediated airway inflammation

**Figure 2:**
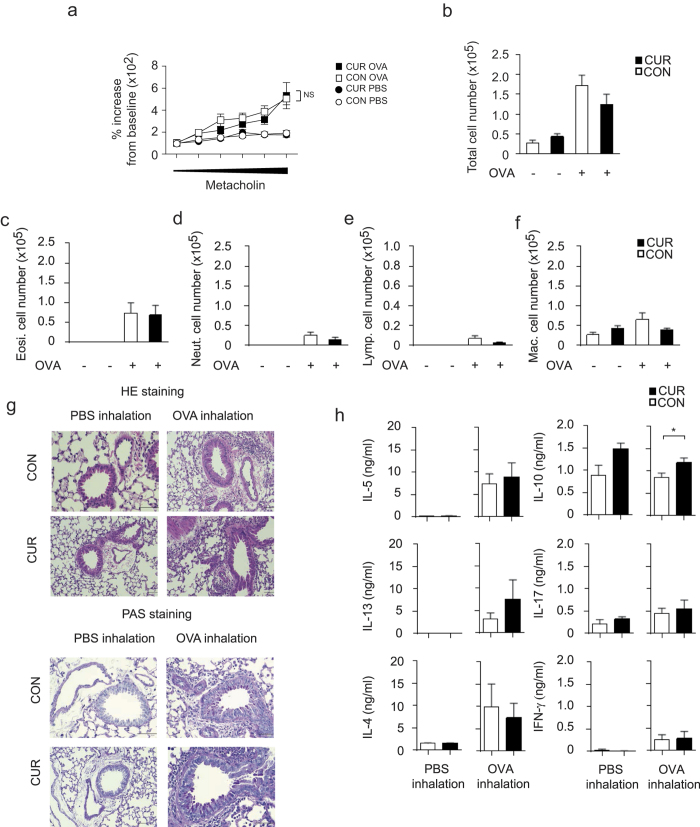
Oral administration of CUR showed little effects on OVA-induced allergic airway inflammation. (**a**) AHR was assessed as lung resistance. Mean values (5 to 6 mice per group) are shown with SEM (square symbols represent OVA challenge, circles represent PBS challenge) (**b**–**f**) The cell number of total mononuclear cells (**b**), eosinophils (**c**), neutrophils (**d**), lymphocytes (**e**), and macrophages (**f**) in the BAL fluid are shown. Mean values with SEM (5 to 6 mice per group) are shown. (**g**) Antigen-induced leukocyte infiltration into the lungs was evaluated by H&E staining (upper panel). Mucus production induced by OVA was examined by histological analysis (PAS staining) (lower panel). (**h**) The concentration of the indicated cytokines in the BAL fluid were measured by cytometric bead array. Mean values with SEM (5 to 6 mice per group) are shown. (**a–f**), no significant differences were found between CUR/OVA and CON/OVA; h, *P < 0.05, Mann-Whitney test).

To study whether oral CUR impairs an on going T cell activation or instead, selectively affects T cell polarization *in vivo*, we next tested the effect of CUR in ovalbumin (OVA)-specific Th2-dominated allergic airway inflammation models. First, airway hyperresponsiveness (AHR) of mice on either CON diet or CUR diet was measured to assess the effect of oral CUR diet on allergic airway inflammation. We found no significant difference in AHR induced by OVA/ALUM immunization followed by OVA inhalation between the mice on CON diet and the mice on CUR diet (Fig. 2a). Consistently, after inhaled antigen challenge, the mice on oral CUR diet showed a similar increase in infiltrating cells including T cells, eosinophils and neutrophils in the bronchoalveolar lavage (BAL) fluid as mice on CON diet (Fig. 2b–f). Histological analysis revealed that CUR diet affected neither the numbers of infiltrated mononuclear cells into the peribronchiolar regions of the lungs (Fig. 2g, upper panel and Supplementary Fig. S3a), nor the production of mucus as shown by periodic acid-Schiff (PAS) staining (Fig. 2g, lower panel and Supplementary Fig. S3b). In accordance with these results, we found equivalent levels of the Th2 cytokines IL-4, IL-5 and IL-13 and enhanced IL-10 in the BAL fluid from the mice challenged with OVA inhalation after CUR diet as in CON-fed mice (Fig. 2h). Altogether, oral administration of CUR showed no suppression of Th2-dominated allergic airway inflammation *in vivo*.

### CUR regulates APC-dependent T cell differentiation

**Figure 3:**
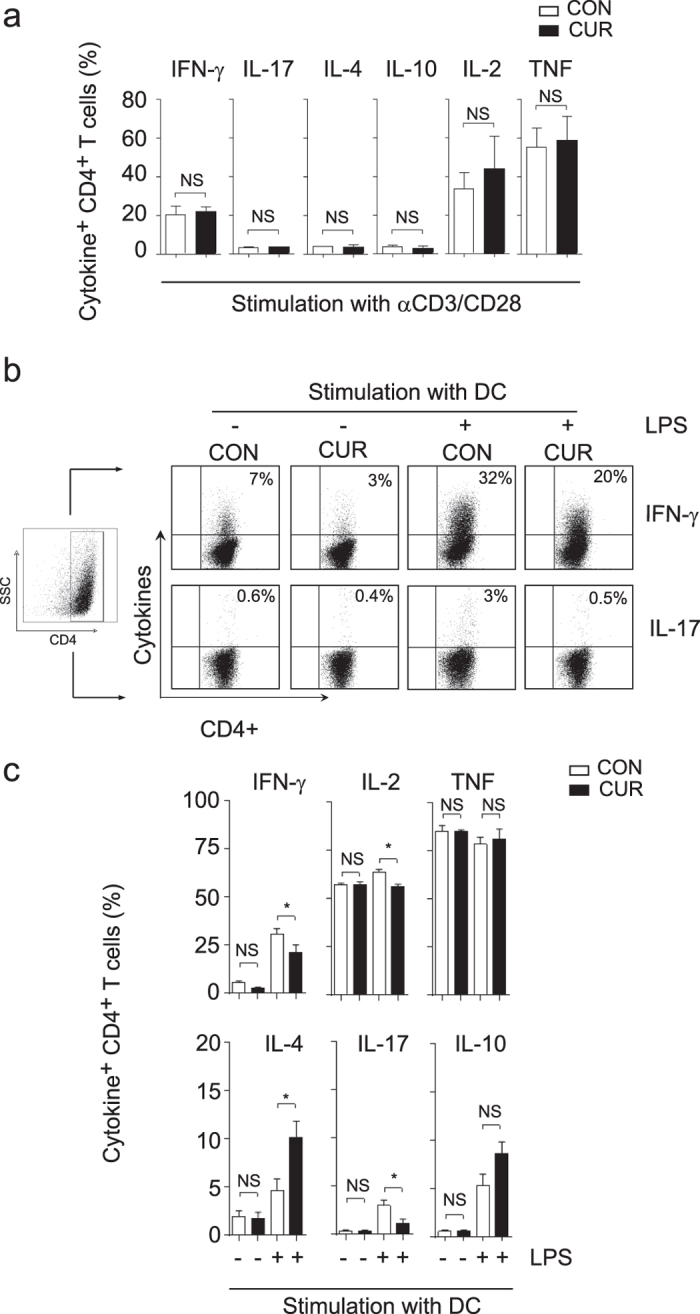
CUR alters the polarization of activated T cells in the presence of DC. (**a**) Isolated CD4^+^ T cells were activated with plate bound anti-CD3 and anti-CD28 Ab and cultivated with DMSO (CON) or curcumin in DMSO (CUR). Cytokine expression was determined after stimulation with PMA/ionomycin by intracellular staining and flow cytometry. Data show mean ± SEM of 4 independent experiments, NS: not significant, Mann-Whitney test. (**b** and **c**) DC were treated with DMSO (CON) or curcumin in DMSO (CUR) for 2 hours, loaded with OVA peptide, left untreated or activated with LPS and used for stimulation of OT II CD4^+^ T cells. On day 7, cells were stimulated with PMA/ionomycin, stained for CD4 and intracellular cytokine production. First, a FSC/SSC plot was made and all leukocytes were gated. The CD4^+^ T cell population was gated by a SSC/CD4 plot (left panel) and then analyzed for CD4^+^ T cell-specific cytokine expression. Dot plots from single experiments are depicted in (**b**), pooled data are shown in (**c**). Bars represent mean ± SEM of three independent experiments (*P < 0.05; One Way ANOVA, Tukey’s *post*-*hoc* test).

The results obtained in the EAE and the allergic airway inflammation *in vivo* models indicate that CUR does not generally suppress antigen-reactive T cell activation but selectively impairs Th17 and Th1 differentiation (Figs 1 to 3). To explore the mechanisms causing these immune-modulating effects of CUR on Th cell polarization we first studied T cell differentiation in an APC-independent system. Therefore we activated freshly isolated CD4^+^ T cells with plate-bound anti-CD3 and anti-CD28 monoclonal antibodies in the presence or absence of CUR. After three days of TCR stimulation and further expansion with IL-2, T cells were analyzed on day seven for the production of IFN-γ, IL-17, IL-4, IL-10, IL-2 and TNF (Fig. 3a). We found no effect of CUR on T cell cytokine production, indicating that the effects of CUR on T cell polarization *in vivo* were not mediated by a direct action of this nutritional on T cells. Since CUR has been reported to impair IL-12 production by APC like DC^13^, CUR may affect T cell differentiation by acting primarily on DC. Non-activated DC were therefore loaded with OVA peptide and co-cultured with OVA-specific T cell receptor-transgenic (OT-II) T cells. On day seven of the co-culture, we found modest expression of IFN-γ, IL-17, IL-4, IL-10 and high production of IL-2 and TNF. The treatment of non-activated DC with CUR had no significant impact on the cytokine phenotype of T cells (Fig. 3b,c). Next, we activated OVA-loaded DC for 1 hour with LPS before initiating the co-culture with OT-II T cells. As expected, LPS-activated DC induced CD4 T cells that were capable to produce large amounts of the lineage-defining cytokines IFN-γ, IL-17 and of IL-4 in OVA-reactive T cells. In sharp contrast, when DC were treated with CUR for 2 hours and subsequently activated with LPS, the DC lost their capacity to strongly induce IFN-γ- and IL-17-producing OT-II T cells, while the induction of IL-4-producing OT-II T cells was significantly enhanced. In contrast to the effects on lineage-defining cytokines, CUR-treated DC had only a minor impact on IL-2, IL-10 or TNF production by OT-II T cells (Fig. 3b,c). The characteristics of the Th cell differentiation induced by CUR with LPS-activated DC *in vitro* reflected those findings on Th cells *in vivo* when mice were immunized in the presence of CUR-containing diet (Fig. 1c).

### CUR selectively inhibits IL-12 and IL-23 production by activated DC

**Figure 4:**
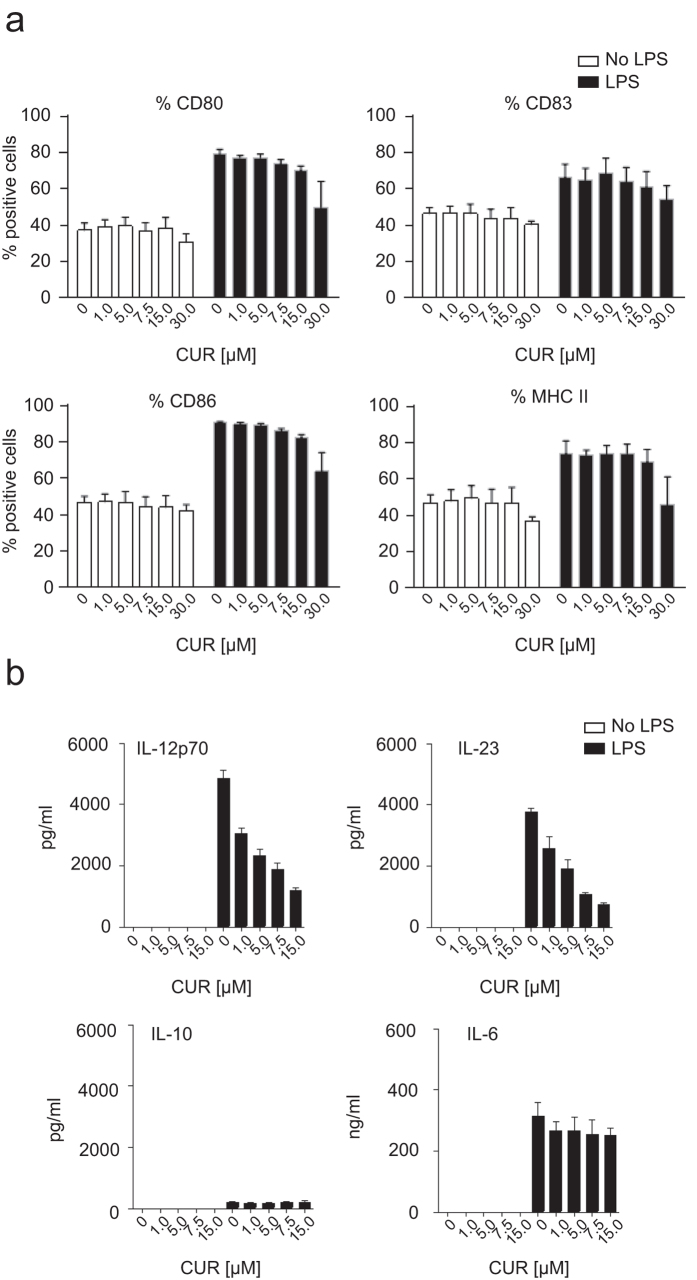
Curcumin affects the DC phenotype without interfering with cell maturation or viability and inhibits IL-12 and suppresses IL-23 production *in vitro*. (**a**) DC were treated with DMSO (CON) or increasing doses of curcumin in DMSO (CUR; 1 to 30 μM) for 2 hours before resting in medium alone or activating with LPS. After 18 hours cells were stained for the expression of CD80, CD83, CD86 and MHC II. Flow cytometry data are depicted as mean ± SEM of 3 independent experiments. (**b**) DC were treated and activated with LPS. Supernatant was collected after 18 hours and analysed for IL-12p70, IL-23, IL-10 and IL-6 secretion by ELISA. The results show mean ± SEM of three biological replicates and represent one out of three independent experiments with similar results.

Since our data and data from other groups argued that CUR affects T cell differentiation by acting on APC^10^,^11^, we explored the effects of CUR on DC differentiation. First, we re-evaluated possible toxic or apoptosis-inducing effects of CUR on DC. CUR concentrations from 1 up to 15 μM did not increase cell death in DC as determined by fixable viability dye staining as well as apoptosis staining with propidium iodide and annexin V (data not shown). Next, we compared the maturation status of DC treated with CUR to control DC and determined the expression of MHC class II and co-stimulatory molecules CD80, CD86 and CD83. As expected, LPS activation induced the up-regulation of MHC class II, CD80, CD86 and CD83 expression by DC. CUR treatment with concentrations ≤15 μM neither affected the maturation status of quiescent DC nor the maturation status of LPS-activated DC (Fig. 4a). From these experiments we concluded that the altered Th cell response by CUR observed *in vitro* and *in vivo* is not due to changes in DC maturation. But, CUR-mediated changes in DC cytokine expression could explain the impaired Th17 and Th1 response. Therefore we first studied the secreted protein levels of IL-12, IL-23, IL-10 and IL-6 after 18 hours of cultivation in medium alone or in presence of LPS. Activation of DC with LPS induced certain amount of IL-12, IL-23 and IL-6 protein, and slight production of IL-10. CUR treatment of non-activated DC did not stimulate cytokine secretion. In sharp contrast, CUR treatment of LPS-activated DC resulted in a dose-dependent suppression of IL-12 and IL-23 production, while the secretion of IL-6 and IL-10 by DC was not affected by CUR treatment (Fig. 4b). Inhibition of IL-12 production from DC by CUR had been reported earlier^11^,^13^. But, even more importantly, we identified that low-dose CUR is also a potent suppressor of IL-23 production by DC. We next aimed to understand the molecular mechanism by which CUR provides an anti-inflammatory phenotype in DC with low expression of IL-12 and IL-23.

### Treatment of DC with CUR enhances STAT3 phosphorylation

**Figure 5:**
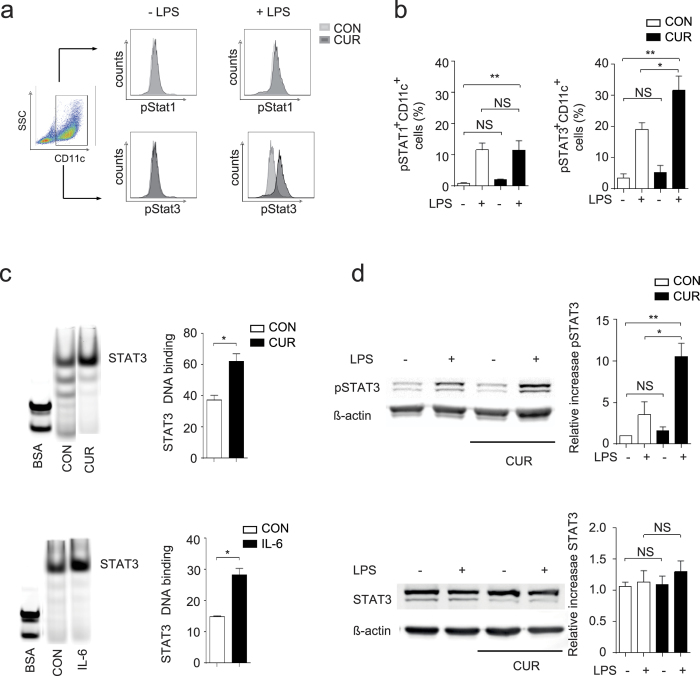
CUR enhances STAT3 phosphorylation in activated DC. (**a,b**) DC were treated with DMSO (CON) or curcumin (CUR) in DMSO (7.5 μM) for 2 hours before activation with LPS for 1 hour. STAT1 and STAT3 phosphorylation was determined by intracellular staining and flow cytometry. First, a FSC/SSC plot was made and all DC were gated. The CD11c^+^ DC population was gated by a SSC/CD11c plot (left panel) and then analyzed for CD11c^+^ DC-specific STAT phosphorylation. Histogram plots from single experiments are depicted in (**a**), pooled data are shown in (**b**). Bars represent mean ± SEM of three independent experiments (*P < 0.05, **P < 0.01). (**c**) EMSA analysis with STAT3 promotor oligonucleotides. DC were treated as in (**a**) or with recombinant IL-6 in medium or medium alone (CON) and then activated with LPS. Nuclei were extracted and mixed with flourochrome labelled STAT3 EMSA probes. Complexes were separated on a TAE-gel and the band intensity was quantified with an OdysseySA Infrared Imaging System. One representative EMSA (left panel) and pooled data from 3 independent experiments (right panel) are shown (mean ± SEM, *P < 0.05; Mann-Whitney test). The samples were run on the same gel, the lanes of interest were cut and re-ordered. (**d**) DC were treated with DMSO (CON) or CUR in DMSO (CUR) as in (**a**) before activation with LPS. Expression of pSTAT3 (upper panel) and total STAT3 protein (lower panel) was analyzed by Western blotting. One representative immunoblot and pooled data from 3 independent experiments are shown. The samples for STAT3/β-actin or pSTAT3/β-actin were run on the same gels and the blot images were cropped based on the molecular weight (79/86 kDa for STAT3/pSTAT3 and 43 kDa for β-actin). Bars represent mean ± SEM of three independent experiments (*P < 0.05, **P < 0.01; One Way ANOVA, Tukey’s *post*-*hoc* test). The complete EMSA (**c**) and Western blot (**d**) figures can be found as Supplementary Figs S7 to S10.

Anti-inflammatory type II DC have been associated with alterations in different signaling pathways^21^. One of these concepts is based on the activation of STAT transcription factors. While the activation of STAT1 in DC is linked to a pro-inflammatory DC phenotype, the activation of STAT3 in DC is associated with an anti-inflammatory or even tolerogenic DC phenotype^22^. We followed the activation status of both STAT1 and STAT3 in our experimental setting since CUR treatment has been suggested to interfere with STAT activation in different cell types^23^. To analyze the balance of STAT1 and STAT3 activation in DC, we quantified the phosphorylated forms of both transcription factors in quiescent and LPS-activated DC. Non-activated DC showed low levels of phosphorylation of both STAT1 and STAT3, irrespective of CUR treatment. As reported, LPS activation induced STAT1 and STAT3 activation in 10–20% of DC^20^,^24^ (Fig. 5a,b). Surprisingly, treatment of DC with CUR and subsequent activation with LPS significantly enhanced phosphorylation of STAT3 without affecting the phosphorylation status of STAT1 (Fig. 5a,b). To investigate if the enhanced phosphorylation of STAT3 by CUR treatment results in functional alteration of the transcriptional activity of STAT3, we quantified the DNA protein binding by STAT3 electromobility shift assay (EMSA). We activated DC with LPS and isolated nuclear extracts, which were tested by EMSA for binding to a STAT3 consensus sequence. As a positive control we treated DC with the STAT3-activating cytokine IL-6 before LPS activation. The nuclear interaction between STAT3-oligos and proteins was weaker in untreated control DC activated with LPS when compared to nuclear extracts from LPS-activated DC that were either treated with CUR or IL-6 (Fig. 5c). These data suggest that CUR significantly increased STAT3 activity in DC after LPS activation. Moreover, Western blot analysis of DC lysates revealed that CUR enhanced LPS-induced STAT3 phosphorylation without affecting total STAT3 protein level (Fig. 5d).

### Activated STAT3 negatively regulates *Il23a* and *Il12b* expression in DC

**Figure 6:**
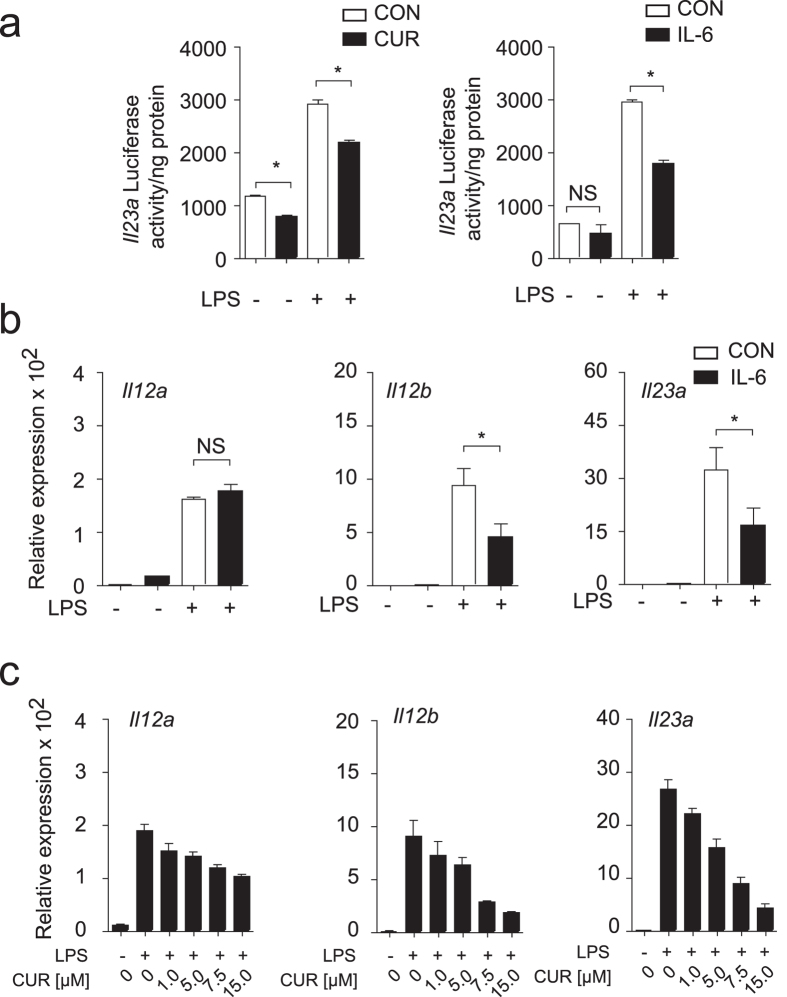
CUR inhibits LPS induced *Il23a* promotor activity. (**a**) Luciferase reporter activity was measured in RAW 264.7 macrophages after 2 hours of incubation or with DMSO (CON) or CUR in DMSO (CUR) and subsequent incubation in the absence or presence of LPS. In addition Luciferase reporter activity was measured in RAW 264.7 macrophages after 1 hour of incubation with recombinant IL-6 (50 ng/ml) or PBS (CON) and subsequent incubation in the absence or presence of LPS. The results show mean ± SEM of quadruplicates. Three independent experiments showed similar results (*P < 0.01; One Way ANOVA, Tukey’s *post*-*hoc* test). (**b**) IL-6 suppresses *Il23a and Il12b* expression in DC after LPS activation. DC were incubated with PBS (CON) or IL-6 (50 ng/ml) and activated with LPS for 1 hour. Expression of the indicated genes was determined by quantitative RT-PCR. The results show mean ± SEM of 4 independent experiments. Data were normalized to ß-actin and expression before LPS activation was set as 1.0 (*P < 0.05; One Way ANOVA, Tukey’s *post*-*hoc* test). (**c**) Treatment of DC with CUR inhibits IL-12 and suppresses IL-23 production *in vitro*. DC were incubated with DMSO (CON) or increasing doses of CUR (1 to 30 μM) in DMSO (CUR) for 2 hours and activated with LPS for 1 hour. Expression of the indicated genes was determined by quantitative RT-PCR. The results show mean ± SEM of 4 independent experiments. Data were normalized to ß-actin and expression before LPS activation was set as 1.0.

We next asked if the enhanced STAT3 activation by CUR is responsible for the regulation of IL-12 and IL-23 in DC. Since activated STAT3 had been reported previously to negatively control *Il12b* promoter activity^25^, we first asked if CUR treatment directly affects *Il23a* promoter activity. To answer this question, we used RAW 246.7 macrophages transfected with an *Il23a* luciferase reporter plasmid. At steady state RAW 246.7 cells showed low basal reporter activity, while LPS activation induced *Il23a* reporter activity. Treatment of macrophages with CUR impaired *Il23a* reporter activity (Fig. 6a). Similar results were obtained, when treating macrophages with the STAT3-activating cytokine IL-6 before LPS activation (Fig. 6a).

To further confirm our findings on the participation of STAT3 in the regulation of IL-23 expression, we next studied the mRNA expression of *Il12a, Il12b* and *Il23a* in DC that were treated with IL-6. Non-activated DC did not express *Il12a, Il12b* or *Il23a* mRNA. IL-6 induced some *Il12a* expression, without affecting *Il12b* or *Il23a* expression. LPS activation of DC strongly induced *Il12a, Il12b* and *Il23a* expression. Treatment of DC with STAT3-activating IL-6 prior to LPS activation significantly inhibited *Il12b* and *Il23a* induction, while *Il12a* expression was not affected (Fig. 6b). We then tested the regulation of IL-12 and IL-23 mRNA expression by CUR. Treatment of DC with increasing doses of CUR prior to LPS activation resulted in a dose-dependent suppression of *Il12b* and *IL23a* expression, but had no significant impact on *Il12a* expression (Fig. 6c). Thus, CUR impairs IL-12 and especially IL-23 protein production by activated DC (Fig. 4b) through inhibition of the transcriptional expression of *Il12b* and *Il23a* (Fig. 6c) and this inhibition is linked to STAT3 phosphorylation (Fig. 5a–c).

**Figure 7:**
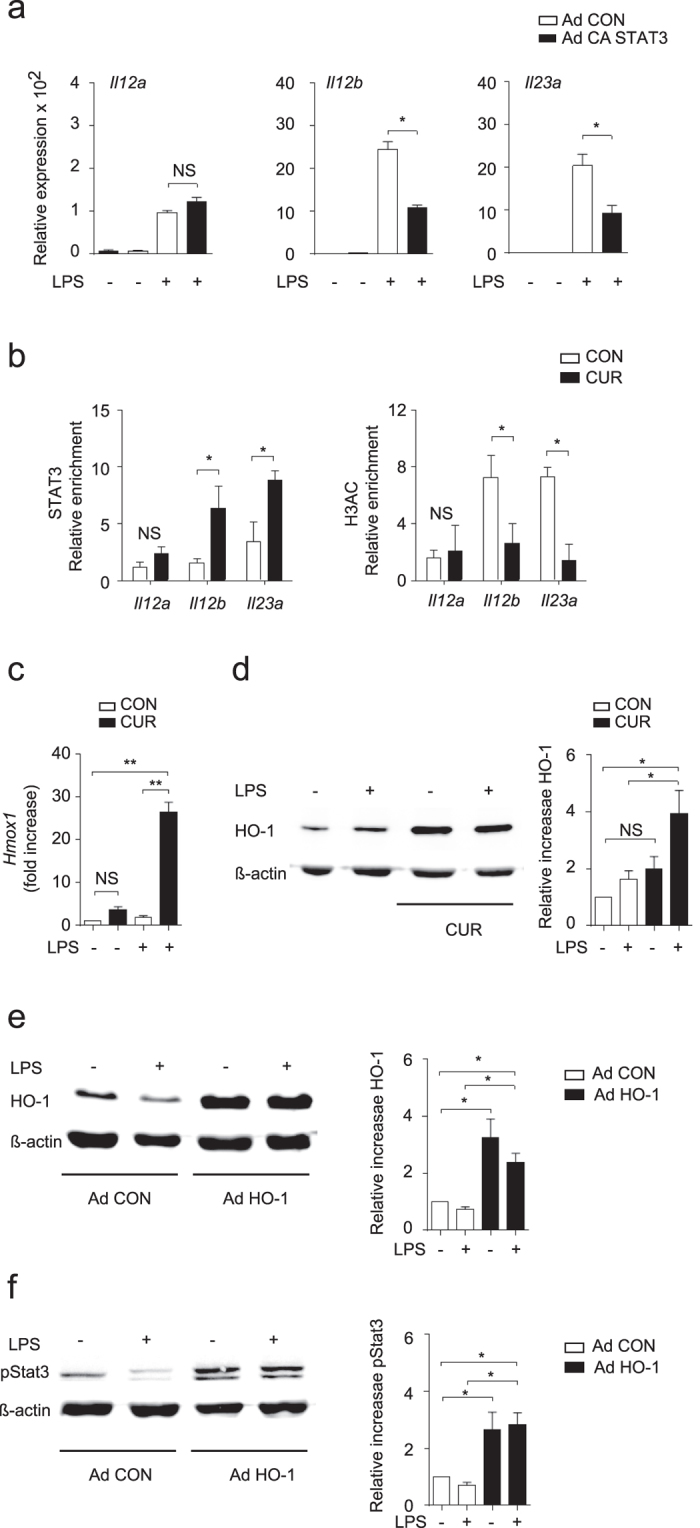
Overexpression of CA STAT3 selectively inhibits *Il12b* and *Il23a* but not *Il12a* expression. (**a**) DCs were infected with control adenovirus (Ad CON) or CA STAT3 (Ad CA STAT3) containing adenovirus and stimulated with LPS for 1 hour. *Il12a, Il12b* and *Il23a* mRNA expression was analyzed by quantitative RT-PCR. Data were normalized to ß-actin and expression before LPS activation was set as 1.0. The results show mean ± SEM of 3 independent experiments (*P < 0.01; One Way ANOVA, Tukey’s *post*-*hoc* test). (**b**) ChIP analysis of DC treated with DMSO (CON) or CUR and stimulated with LPS, followed by crosslinking and immunoprecipitation with antibody to STAT3 (left panel) or acetylated histone H3 (H3Ac, right panel). Bound DNA was amplified by quantitative PCR for primer sites in *Il12a, Il12b* and *Il23a* promoter regions. Bars represent mean ± SEM of 3 independent experiments (*P < 0.05; One Way ANOVA, Tukey’s *post*-*hoc* test). (**c**) HO-1 is induced by CUR in DC *in vitro*. DC were treated with DMSO (CON) or CUR in DMSO (CUR) before activation with LPS for 1. Expression of *Hmox1* mRNA was determined by quantitative RT-PCR (**c**) and HO-1 protein was analyzed by Western blotting (**d**). The results in (**c**,**d**) show mean ± SEM of three independent experiments (*P < 0.05, **P < 0.01; One Way ANOVA, Tukey’s *post*-*hoc* test), the blot shown in (**d**) is representative for three independent experiments with similar results. Actin expression served as control. (**e**,**f**) Overexpression of HO-1 increased STAT3 phosphorylation in DC. DCs were infected with control adenovirus (Ad CON) or HO-1 containing Ad HO-1 and stimulated with LPS for 1 h. Overexpression of HO-1 protein was analysed by Western blotting (**e**). The expression of phosphorylated STAT3 (pSTAT3) in DC treated as in (**e**) was analysed by Western blotting (**f**). One representative immunoblot and pooled data from 4 independent experiments are shown (mean ± SEM *P < 0.05; One Way ANOVA, Tukey’s *post*-*hoc* test). The Western blot samples for HO-1/β-actin or pSTAT3/β-actin were run on the same gels and the blot images were cropped based on the molecular weight (32 kDa for HO-1, 79/86 kDa for pSTAT3 and 43 kDa for β-actin). The complete Western blot figures (**d**–**f**) can be found as Supplementary Figs S11 to S13.

To further prove that STAT3 directly regulates *Il12b* and *Il23a* expression independently of DC cytokine production or CUR treatment, we infected DC with adenoviral vectors containing a control construct (Ad CON) or construct encoding constitutive active STAT3 (Ad CA STAT3) prior to LPS activation. Neither Ad CON nor Ad CA STAT3 constructs influenced IL-12 or IL-23 expression in non-activated DC (Fig. 7a). Cells treated with Ad CON and activated with LPS showed strong induction of *Il12a, Il12b* and *Il23a*. Importantly, Ad CA STAT3 significantly suppressed *Il12b* and *Il23a* expression of LPS-activated DC, while *Il12a* was not affected (Fig. 7a).

To further assess the direct regulation of IL-12 and IL-23 by CUR-activated STAT3, we performed chromatin immunoprecipitation (ChIP) using DC lysates and antibodies directed against STAT3 or histone 3 acetylation (H3Ac). We found enhanced binding of STAT3 to the *Il12b* and *Il23a* promoter in LPS-treated DC treated with CUR compared to DC treated with DMSO (CON) (Fig. 7b). The binding of STAT3 to the *Il12a* locus was not significantly affected by CUR treatment. Likewise, we found significantly less H3Ac within the *Il12b* and *Il23a* promoter regions but not the *Il12a* promoter region (Fig. 7b). Thus, CUR negatively regulated *Il12b* and *IL23a* expression through enhanced STAT3 activity. By using a pharmacological STAT3 inhibitor we could abolish the negative regulation of CUR on *Il12b* and *IL23a expression in* RAW 246.7 macrophages activated with LPS (Supplementary Fig. S4).

### CUR enhances STAT3 phosphorylation through induction of HO-1

**Figure 8:**
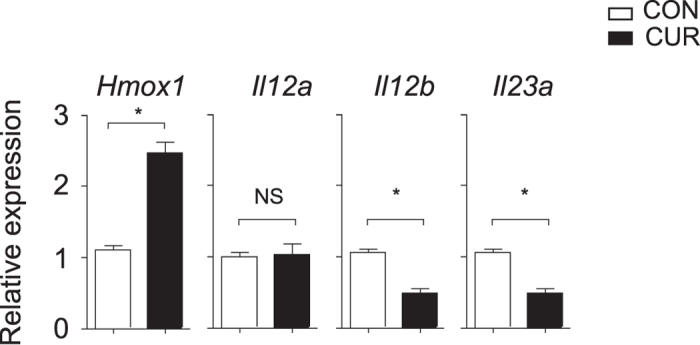
CUR induces *Hmox1* and suppresses *Il12b* and *Il23a* expression *in vivo*. Mice were fed with CUR or a control diet (CON) and immunized for EAE. On day three after immunization draining lymph nodes were isolated and expression of *Hmox1, Il12a, Il12b* and *Il23a* was determined by quantitative RT-PCR. Bars represent mean ± SEM of control mice (n = 5) and CUR-fed mice (n = 5). Data were normalized to ß-actin (*P < 0.05, NS: not significant, Mann-Whitney test).

We further decided to unravel the mechanism by which CUR enhances STAT3 phosphorylation in LPS-activated DC. Since IL-6 and IL-10 production were minimally affected by CUR (Fig. 4b), a cell-intrinsic effect could explain the enhanced STAT3 activity. The expression of Src homology phosphatase-1 (SHP-1), a protein tyrosine phosphatase with negative impact on STAT3 activity, was not affected by CUR in LPS-activated DC (Supplementary Fig. S5). A well-documented effect of CUR is the induction of heme oxygenase 1 (HO-1) in various cell types and HO-1 has been reported to interact with the activity of transcription factors including STAT proteins^26^. Therefore, we investigated the regulation of HO-1 in DC by CUR treatment. Quantitative analysis showed that CUR enhanced *Hmox1* mRNA and HO-1 protein expression in DC, especially after activation with LPS (Fig. 7c,d). To evaluate possible effects of HO-1 on STAT3 activation we overexpressed HO-1 by an adenoviral construct in DC (Fig. 7e) and studied the levels of phosphorylation of STAT3 by Western blotting. Strikingly, Ad HO-1 but not Ad CON transfection resulted in enhanced STAT3 phosphorylation, independently of LPS activation (Fig. 7f). To translate these findings to the *in vivo* situation we studied the expression of HO-1, IL-12 and IL-23 in draining lymph nodes of mice immunized for EAE on day 3. Mice on oral CUR diet showed a significant induction of *Hmox1* expression and suppressed levels of *Il12b* and *Il23a*, while *Il12a* expression was not affected by CUR (Fig. 8).

Taken together, we could show that the nutritional CUR negatively regulates *Il12b* and *Il23a* expression through HO-1 and STAT3 interaction (Supplementary Fig. S6). Our study shows how low concentrations of a classical nutritional factor can influence molecular events that link anti-oxidative and anti-inflammatory signalling pathways, and, that help to control T cell-mediated autoimmunity.

## Discussion

Numerous studies have highlighted the beneficial effects of dietary CUR as anti-tumoral and anti-inflammatory phytochemical^8^,^9^,^10^,^11^,^12^,^13^,^14^,^15^,^16^. Mechanistically, CUR has been reported to interact with multiple signaling pathways^18^,^19^. Many of the molecular and cellular interactions observed, require high concentrations of CUR and some of these interactions are associated with toxic or apoptotic effects. For instance CUR at doses of 50 μM has been reported to inhibit NF-κB activation but in addition induces apoptotic cell death in myeloma cell lines studied. In contrast, CUR at low concentrations (≤10 μM) did not show such effects in this setting^27^. Similar results have been observed in other cell types treated with CUR *in vitro* demonstrating that only low concentrations of CUR (≤10 μM) allow study specific molecular events that are not linked to apoptotic pathways or loss of cell viability. This is of importance, since the *in vivo* absorption of dietary CUR is low and the tissue levels achieved are around 0.5 to 3 μM^28^,^29^. In clinical studies CUR is administrated orally using capsules, solid lipid nanoparticles or complex formulations^30^.

Here, we show that oral CUR diet prevents severe encephalomyelitis in mice, a model that strictly depends on IL-23 and Th17 responses^31^. The data on the clinical EAE parameters are in agreement with other reports demonstrating that injections of CUR improve EAE in rodents^13^,^14^,^15^,^16^. Though, we did not find any beneficial effects of dietary CUR in a Th2-dominated asthma model using OVA. Other groups have revealed an attenuation of airway inflammation in mice by CUR^32^,^33^,^34^. The discrepancy of the results between our study and others could be caused by the different route of CUR administration (intranasal or intraperitoneal administration versus oral feeding) and by the different protocols used for the induction of airway inflammation (repetitive OVA exposure and chronic airway inflammation versus acute airway inflammation)^32^,^33^. The airway inflammation model used by us is less sensitive to IL-23, a cytokine critically affected by oral CUR administration.

The main immune cell population directly affected by CUR seems to be the DC population. In our studies, CUR concentrations up to15 μM had no effect on DC surface receptors. Other reports demonstrate that CUR concentrations of ≥20 μM impair the expression of costimulatory molecules by DC^17^,^35^,^36^ and as a consequence inhibit their capacity to stimulate T cell proliferation^17^,^35^,^36^,^37^. Since in our experimental setting, CUR concentrations ≥20 μM started to affect DC viability, we used low CUR concentrations that neither affected DC viability nor DC maturation (Fig. 4). Importantly, no suppressive effects on T cell proliferation have been observed when using DC treated with CUR ≤ 10 μM *in vitro*^10^ nor by oral CUR diet *in vivo* (Figs 1b and 2b). This is in agreement with a report demonstrating that CUR supplemented in drinking water does not affect antigen-specific T cell proliferation in mice immunized for EAE^38^.

In this study, we found that low CUR concentrations equip DC with an anti-inflammatory profile. Treatment of DC with 7.5 μM CUR specifically suppressed the expression of LPS-induced IL-12p40 and IL-23p19 (Figs 4b and 6c) and by this attenuated inflammatory Th17 responses and instead promoted Th2 cell development (Fig. 1d). In the human setting dietary supplementation of CUR could be beneficial for patients with IL-23/Th17-dominated inflammatory autoimmune disease like multiple sclerosis. To unravel the molecular mechanisms we focused on signaling pathways that had been linked to CUR and to the inflammatory status of DC. One of these pathways is the NF-κB pathway, which is also implicated in the regulation of *Il12b* and *Il23a*^20^,^39^. *In vitro*, CUR concentrations ≥25 μM have been reported to affect NF-κB activity in activated DC and macrophages^17^,^40^,^41^. However, *in vivo*, CUR showed no direct effects on NF-κB activity, as demonstrated by using NF-κB-driven reporter gene transgenic mice under inflammatory conditions^40^,^41^. One other signaling pathway linked to the effects of CUR is the JAK/STAT pathway. *In vitro*, CUR has been reported to modulate cytokine-dependent STAT4 phosphorylation in human T cells and to inhibit STAT3 phosphorylation in multiple cell types when using doses of 20 to 50 μM^13^,^23^,^42^,^43^,^44^. As mentioned above, the concentrations used to study the effects of CUR on signaling pathways are critical, since concentrations of CUR ≥ 20 μM can impair cell viability and induce apoptosis^43^,^44^. To our surprise, we found enhanced STAT3 activation in DC treated with CUR < 10 μM, which resulted in a negative regulation of *Il12b* and *Il23a* in LPS-activated DC. Activated STAT3 has been reported previously to impair *Il12b* gene expression and IL-12 protein secretion by LPS-activated DC^24^,^25^,^45^,^46^,^47^. A similar mechanism seems to be valid for *Il23a*, but not for *Il12a* (Fig. 7a). Here we show that STAT3 binds to the promoter loci of *Il12b* and *Il23a*, suppresses the transcription of *Il12b* and *Il23a* accompanied with the negative regulation of H3Ac (Fig. 7b). DC themselves produce STAT3-activating cytokines like IL-6 or IL-10 upon LPS activation. We did not find significant changes in IL-6 or IL-10 secretion by CUR-treated DC *in vitro*, indicating that the mechanism of enhanced STAT3 activation by low-dose CUR is cell-intrinsic.

One of the molecular events readily induced by even minimal concentrations of CUR (2 μM) that can also be achieved *in vivo* is the induction of HO-1 as demonstrated in various cell types including macrophages^48^. We found a rapid induction of *Hmox1* mRNA and HO-1 protein in DC by low-dose CUR (Fig. 7c,d). The expression of HO-1 is regulated by Nrf2^48^,^49^ and CUR activates this signaling pathway. Whole genome expression profiling unraveled that oral CUR feeding regulates more than 800 NRF2-dependent genes *in vivo* with a >2-fold induction or suppression. Among these genes *Hmox1* was one of the most highly induced (>75-fold induction in the small intestine) by oral CUR^50^. Likewise we found a significant increase of *Hmox1* in draining lymph nodes of mice on CUR diet after immunization for EAE (Fig. 8).

As reported previously, HO-1 translocates into the nucleus where it interacts with the activity of classical transcription factors including NF-κB and STAT3^5^,^20^,^26^, which are critically implicated in the regulation of *Il12b* and *Il23a* expression in DC and macrophages^25^,^51^. Here, we could identify that a nutritional affects IL-23-dominated autoimmune disease by influencing the HO-1/STAT3 axis. CUR induced HO-1 expression and STAT3 phosphorylation in DC resulting in an anti-inflammatory DC phenotype. By overexpression of HO-1 in DC, we could show an enhancement of STAT3 phosphorylation (Fig. 7f). This mechanism is also relevant *in vivo*. Recent studies demonstrated that feeding CUR enhances STAT3 activation as shown in rodent models of chemically-induced colitis^52^ and ischemia-reperfusion injury^53^,^54^. Nrf2/HO-1 is a key factor mediating the immune-regulatory properties of CUR and other Nrf2 activators^5^,^20^. The anti-inflammatory properties of HO-1/STAT3 interaction seem to be of general importance. As demonstrated recently, overexpression of HO-1 in Nrf2-deficient macrophages promotes STAT3 signaling and anti-inflammatory effects in a mouse model of liver ischemia/reperfusion injury^55^. Interestingly, the regulating effects of HO-1 on STAT3 activity seem to be cell-type specific. In keratinocytes, HO-1 activation by hemin or cobalt protoporphyrin inhibits STAT3 activity through the induction of SHP-1^56^. This is different in DC, where HO-1 induction by CUR enhances STAT3 activity without affecting SHP-1 expression (Fig. 5 and Supplementary Fig. S5).

Taken together we could unravel the mechanism by which two proposed molecular targets of the nutritional CUR, namely HO-1 and STAT3 interact and determine the phenotype of DC controlling IL-23/Th17 responses (Supplementary Fig. S6).

## Methods

### Mice

SJL mice, 6–8 weeks of age, were purchased from Janvier, C57BL/6 mice, were purchased from Charles River or CLEA and maintained in the animal laboratories of the FORS Institute, University of Tübingen or in the animal facility of Chiba University. Animal experiments were approved by the Institutional Animal Care and Use Committee of the Regierungspräsidium Tübingen (HT01/08 and HT10/13) and the Chiba University Review Board for Animal Care. All animal procedures and methods were carried out in accordance with the approved guidelines and institutional regulations.

### Experimental autoimmune encephalomyelitis

EAE was induced by subcutaneous immunization of female SJL mice with 37.5 μg PLP_139-151_ Peptide (Genaxxon bioscience) in Complete Freund’s Adjuvant (CFA) (Difco) and i.p. injection of 200 ng pertussis toxin (Calbiochem)^20^. SJL mice were fed with 2% curcumin (CUR)-containing food pellets or received a control diet (CON), starting 10 days prior to immunization. CUR (Sigma-Aldrich) was mixed into food pellets by a professional animal food manufacturer (Ssniff). CNS-infiltrating mononuclear cells were isolated on day 18 after immunization by using a commercial neural tissue dissociation kit (Miltenyi Biotec), followed by a Percoll^®^ (Sigma-Aldrich) density gradient centrifugation, trypan blue staining and cell quantification using a Neubauer chamber. The clinical EAE score was followed and rated by the following scale: 0 - no disease; 1 - limp tail; 2 - hind limb weakness or partial paralysis; 3 - complete hind limb paralysis; 4 - fore limb and hind limb paralysis; 5 - moribund state^20^,^57^.

### Airway inflammation model

C57BL/6 mice were fed with 2% CUR-containing food pellets or received a control diet from day 0. The mice were immunized with 250 μg OVA (Sigma-Aldrich) i.p. in 4 mg of aluminum hydroxide gel (alum) on days 8 and 15. The mice were nebulized with aerosolized 1% OVA in saline for 30 minutes using a supersonic nebulizer (model NE-U07; Omron) on days 22 and 24. Airway hyper responsiveness was assessed by measuring the changes in lung resistance in response to increasing doses of inhaled methacholine, as described previously^58^. For collection and anaylsis of bronchioalveolar lavage (BAL) fluid the lungs were lavaged with 1.0 ml of PBS one day after the last OVA inhalation (day 25). For the differential cell count, BAL fluid was cyto-centrifuged onto slides by a Cytospin 4 (Thermo Electron) and stained with Diff-Quick (Sysmex). For histology, the lung samples taken on day 25 and sections were stained with H&E and PAS reagents as previously described^58^. Slides were examined in a random blinded fashion. The numbers of infiltrated inflammatory cells at the peribronchial region were counted as visible on H&E stained lung sections. Infiltration of inflammatory cells in the lung was graded with a semiquantitative scoring system (5 = large widespread infiltrate around the majority of vessels and bronchioles to 1 = small number of inflammatory foci). Goblet cells were counted on Periodic Acid-Schiff (PAS)-stained lung sections. Percentage of PAS positive cells were calculated in each mouse (n = 3 in each group). The goblet cell hyperplasia was graded with a semiquantitative scoring system (0 = <5% goblet cells in airway epithelium; 1 = 5–25%; 2 = 25–50%; 3 = 50–75%; 4 = >75%) as described^59^. The average of the airway scores from each lung was expressed as mucus score in arbitrary units.

### RNA isolation and gene expression

Total RNA was purified from cultured cells or from *ex vivo* isolated cells and reverse transcribed into cDNA using commercially available kits (Biozym). Relative gene expression levels were determined by quantitative realtime-PCR (qRT-PCR) using TaqMan probes (TIB MolBiol) for *ßactin, Il6, Il10, Il12a, Il12b, Il23a* and *Hmox1* and the LightCycler480 system (Roche). The *relative expression* of the indicated genes was calculated relative to the expression of *ß*-*actin*. Control conditions were set as 1.0 as indicated.

*ßactin* for: ACCCACACTgTgCCCATCTA, rev: gCCACAggATTCCATACCCA

TM: 6FAM-CATCCTgCgTCTggACCTggC-BBQ

*Il6* for: CggAggCTTAATTACACATgTTCTC, rev: ggTAgCTATggTACTCCAgAAgACCA

TM: 6FAM-ACgATgATgCACTTgCAgAAAACAATCTgA-BBQ

*Il10* for: AgCTggACAACATACTgCTAAC, rev: CTCTTATTTTCACAggggAGAA

TM: 6FAM-CgCCTCAgCCgCATCCTGAgggTC-BBQ

*Il12a* for: CATggTgAAgACggCCAgA, rev: CCAggCAACTCTCgTTCTTgT

TM: 6FAM-AggTCTTCAATgTgCTggTTTggTCCC-BBQ

*Il12b* for: gCTCAgAgTCTCgCCTCCTT, rev: gAgCTggAgAAAgACgTTTATgTTg

TM: 6FAM-ACATggAgTCATAggCTCTggAAAgACCC-BBQ

*Il23a* for: CACCAgCgggACATATgAATC, rev:CAgAACTggCTgTTgTCCTTgA

TM: 6FAM-CACTggATACggggCACATTATTTTTAgTC-BBQ

*Hmox1* for: TgCTCgAATgAACACTCTggAgA, rev: gTTTCCCTCggggTgTCT

TM: 6FAM-ACgAAgTgACgCCATCTgTgAgggA-BBQ

*Ptpn6* for: ggTgTCCTCAgCTTTCTggAT, rev: AATgTCACAgTCTAgCCCCTTg

TM: 6FAM-CCCATCATTgTgCATTgCAgCg-BBQ

### Cytokine analysis and flow cytometry

Commercially available ELISA Kits were used for the quantification of the cytokines IL-12p70, IL-23, IL-6 and IL-10 from cell culture supernatants. Immune cells were isolated from draining lymph nodes of immunized mice on day 3 or 7 as indicated and analzsed for cytokine production by qRT-PCR or flow cytometry. Intracellular cytokine staining was performed after stimulating cells with PMA and ionomycin (Sigma-Aldrich) in the presence of GolgiStop^®^ (BD Biosciences) for 4 h^20^. Cells were fixed with 2% formaldehyde, permeabilized with saponin containing buffer and stained with fluorochrome-labeled antibodies directed against CD4 (Gk1.5, Biolegend) and IFN-γ (XMG1.2, eBioscience), IL-4 (11B11, eBioscience), IL-17A (TC11-18H10, BD Biosciences), IL-2 (JES6-5H4, eBioscience), IL-10 (JES5-16E3, eBioscience) or TNF (MP6-XT22, eBioscience). Fluorochrome-labeled anti-CD11c (HL3, BD Biosciences) antibodies were used for staining DC. Cells were analysed by flow cytometry (LSRII fow cytometer, BD Biosciences) and collected data were analysed by FLOWJO/FCS Express. Concentrations of indicated cytokines in BAL fluid were measured by Cytometric Bead Array (BD Biosciences, San Jose, CA, USA).

### Cell culture

Bone marrow-derived DC were isolated and cultivated as described previously^57^,^60^. DC were harvested on day 7, stimulated with CUR (Sigma-Aldrich) in DMSO or DMSO alone as control and activated with 100 ng/ml LPS from *E. coli* O111:B4 (Sigma-Aldrich) for the indicated time points. CD4^+^ T cells were isolated from spleen and lymph nodes of naïve C57BL/6 mice by using CD4 microbeads and MACS^®^ purification system (Miltenyi Biotec). Isolated T cells were activated for 3 days with 10 μg/ml plate-bound anti-CD3 and anti-CD28 antibodies (eBiosciences) with DMSO or 7.5 μM CUR in DMSO and expanded for 4 days with IL-2 (Chiron Therapeutics). For co-culture experiments, DC were first treated with 7.5 μM CUR in DMSO or DMSO for 1 h, left untreated or activated with 100 ng/ml LPS and loaded with 100 ng/ml ovalbumin (OVA_323-339_)-peptide (EMC microcollections) for another 1 h. Then, purified CD4^+^ T cells isolated from OT-II mice were cultured with these DC. On day 7 cytokine expression by T cells was determined after activation by intracellular staining and flow cytometry using a LSRII flow cytometer (BD Biosciences).

### *Il23p19* reporter assay

RAW 264.7 macrophages were transfected with a pGL3-*Il23p19* reporter plasmid (bp −1180/+110 promoter fragment) using Lipofectamine^TM^ 2000 (Invitrogen). The p19 luciferase construct was a gift from Y. Chen (University of Pennsylvania, Philadelphia, PA)^61^. Twenty-four hours post transfection cells were pre-treated with 7.5 μM curcumin in DMSO or DMSO alone for 1 h and activated with 100 ng/ml LPS. After 6 h cells were lysed with passive lysis buffer (Promega) and luciferase activity was measured using a luminescence microplate reader (Berthold Technologies). Protein concentration of each sample was determined by a protein assay (Roth) and luciferase activity was calculated in relation to the protein content.

### Western blotting

DC were incubated with 7.5 μM curcumin in DMSO or DMSO alone and activated with 100 ng/ml LPS for the indicated time points. Cells were washed in ice-cold PBS, lysed and heated in SDS sample buffer [125 mM Tris-HCl (pH 6.8), 2% w/v SDS, 10% glycerol, 100 mM DTT, and 0.01% w/v bromphenol blue] at 95 °C for 5 min. Equal amounts of samples were loaded onto a 12% PAA gel and transferred to a PVDF membrane or to a nitrocellulose membrane. After blocking with blocking buffer for 1 h the membrane was incubated with anti-actin (Millipore), anti-pStat3, anti-Stat3, anti-SHP-1 (Cell Signaling Technology) or anti-HO-1 (Enzo Life Sciences) antibodies overnight at 4 °C, followed by incubation with IRDye680RD or IRDye800CW-conjugated secondary antibody (Licor) for 1 h. Proteins were detected by OdysseySA Infrared Imaging System (Licor). The band size was quantified using the OdysseySA software version 1.1. Western blot images were cropped based on the molecular weight of indicated proteins.

### Overexpression of HO-1 and STAT3

E1- and E3-deleted adenoviral vectors (Ad) were expressing EGFP, CA STAT3 or mouse HO-1-EGFP were gifts from T. Tüting (University of Bonn, Germany), H. Inoue (Kanazawa University, Ishikawa, Japan) and J.W. Kupiec-Weglinski (University of California, Los Angeles, USA). All recombinant adenoviruses were based on Ad5 and contained the transgene under the control of the CMV immediate–early promoter. Viruses were propagated in 293 cells, purified by cesium chloride density gradient centrifugation and subsequent dialysis according to standard protocols, and stored at 70 °C. Adenoviruses were added to DC cultures on day 8 at a multiplicity of infection of 300. Cells were incubated for 24 h at 37 °C and 7.5% CO2 before stimulation with LPS for indicated time points. Infection efficiency was confirmed by flow cytometry and Western blot analysis of HO-1. For inhibition of STAT3 activity in RAW 264.7 macrophages a pharmacological inhibitor (STAT3 inhibitor V, Calbiochem) was used.

### Chromatin immunoprecipitation (ChIP)

ChIP was performed as described previously^20^. In brief, DC were treated with curcumin in DMSO (7.5 μM) or DMSO alone for 1 h and activated with 100 ng/ml LPS for 1.5 h. Cells were cross-linked, harvested, and lysed by sonication. Subsequently, DC lysates were immunoprecipitated with anti-Stat3 (Cell Signaling Technology) or anti-acetyl histone H3 (Millipore) antibodies. The precipitated and eluted DNA was analyzed by qRT-PCR. Primers were designed to amplify 150–250 bp fragments from selected genomic regions. The qRT-PCR was performed in duplicate on each sample and input DNA using LightCycler480 (Roche) and TaqMan probes (TIB MolBiol) according to the manufacturers’ instructions. To account for differences in DNA quantity, for every genomic sequence studied, a ΔCt value was calculated for each sample by subtracting the Ct value of the chromatin-immunoprecipitated sample from the Ct value obtained for the input and no antibody ChIP, respectively. Calculating 2^ΔΔCt^ yielded the relative amount of PCR product (relative enrichment). Binding sites for Stat3 within the *Il23a, Il12b* and *Il12a* promoter regions were identified with Matinspector (Genomatix). Primers encompassing putative STAT3-binding sites in the indicated promoter regions were designed by TIB MolBiol.

*Il23a* for: AggCAggTggATTTCTgAgTTC, rev: ggTTgCTCTTCCAgAggTCCT

TM: 6FAM-CAgCCAAggCTATACAgAgAAACCCTgT-BBQ,

*Il12b* for: gggCCTgTAACACCTACTTATTTgA; rev: ggCTAACCTCTCCCgATTCC

TM: 6FAM-ACCgAgTTCATggTTCATgCCACA-BBQ,

*Il12a* for: TTTTAAgACAAgCTCTCATgTCATTg,

rev: TAgATgCTgAgCCAgTgATgAgTACA

TM: 6FAM-AgCTATgTTTATggCTgAggCACAAgTTCA-BBQ

### T cell proliferation assay

For determining proliferation of *in vivo* activated T cells during EAE, CD4^+^ cells were isolated from draining lymph nodes of immunized mice and co-cultured with PLP-pulsed DC for 3 days. ^3^H-thymidine (0,25 μCi/well; PerkinElmer) was added for 16 h prior to harvest. Incorporation of radioactivity was determined by scintillation counting. Data are shown as means of triplicate cultures. The standard deviation was less than 10% of the mean cpm.

### Electrophoretic Mobility Shift Assay

DC were treated with curcumin in DMSO (7.5 μM) or DMSO alone for 1 h and activated with 100 ng/ml LPS for 1 h. Nuclei were extracted using nuclear and cytoplasmic extraction reagents (Thermo Scientific). The binding mixture (20 μl) consisting of 5 μg protein of nuclear extract, 25 mM DTT, 2,5% Tween-10, 1%NP40, 100 mM MgCl_2_, 1 μg poly(di-dC) and 10 ng of a IRDye 700 STAT3 Consensus Oligonucleotide was incubated for 1 h at RT. The DNA-protein complexes were separated on native 15% TAE and detected by a OdysseySA Infrared Imaging System (Licor). The band intensity was quantified using the OdysseySA software version 1.1 (Licor).

### Statistics

All data were analyzed and plotted using GraphPad Prism 6 software. Statistical analyses were performed by using the Mann-Whitney test, the Wilcoxon test or the Tukey’s *post*-*hoc* test after One-way ANOVA as indicated. Values of *P* < 0.05 were considered significant.

## Additional Information

**How to cite this article**: Brück, J. *et al*. Nutritional control of IL-23/Th17-mediated autoimmune disease through HO-1/STAT3 activation. *Sci. Rep.* **7**, 44482; doi: 10.1038/srep44482 (2017).

**Publisher's note:** Springer Nature remains neutral with regard to jurisdictional claims in published maps and institutional affiliations.

## Supplementary Material

Supplementary Information and Data
