# Supplementary material for: Nutritional control of IL-23/Th17-mediated autoimmune disease through HO-1/STAT3 activation
Source: Sci Rep. 2017 Mar 14;7:44482. doi: 10.1038/srep44482 (PMC5349589; doi:10.1038/srep44482)

**Supplementary Information**

**Nutritional control of IL-23/Th17-mediated autoimmune disease**

**through HO-1/STAT3 activation**

Jürgen Brück^1^, Julia Holstein^1^, Ivana Glocova^1^, Ursula Seidel^1^, Julia Geisel^1^, Toshio Kanno^2^, Jin Kumagai^2^, Naoko Mato^2^, Stephan Sudowe^3^, Katja Widmaier^1^, Tobias Sinnberg^1^, Amir S. Yazdi^1^, Franziska C. Eberle^1^, Kiyoshi Hirahara^2^, Toshinori Nakayama^2^, Martin Röcken^1^ and Kamran Ghoreschi^1^

^1^Department of Dermatology, University Medical Center of the Eberhard Karls University Tübingen, 72076 Tübingen, Germany

^2^Department of Advanced Allergology of the Airway, University of Chiba, Chiba 260-8670, Japan

^3^Department of Dermatology, University Medical Center of the Johannes Gutenberg University Mainz, 55101 Mainz, Germany

Correspondence:

Dr. Kamran Ghoreschi

Department of Dermatology, University Medical Center

Eberhard Kalrs Unviversity Tübingen, Germany

Phone: +49 7071 29 80816

E-mail: [kamran.ghoreschi@med.uni-tuebingen.de](mailto:kamran.ghoreschi@med.uni-tuebingen.de)


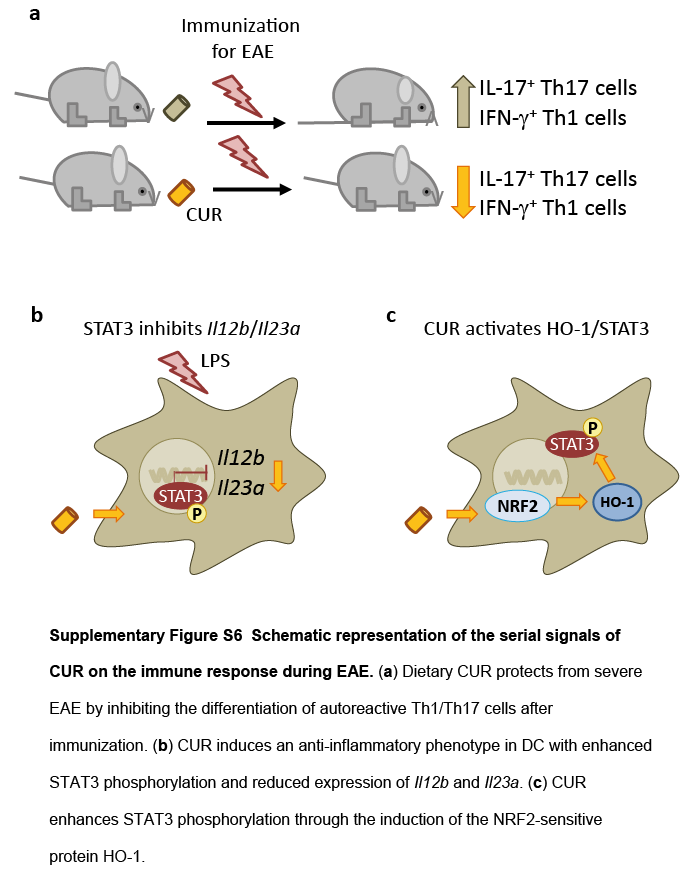


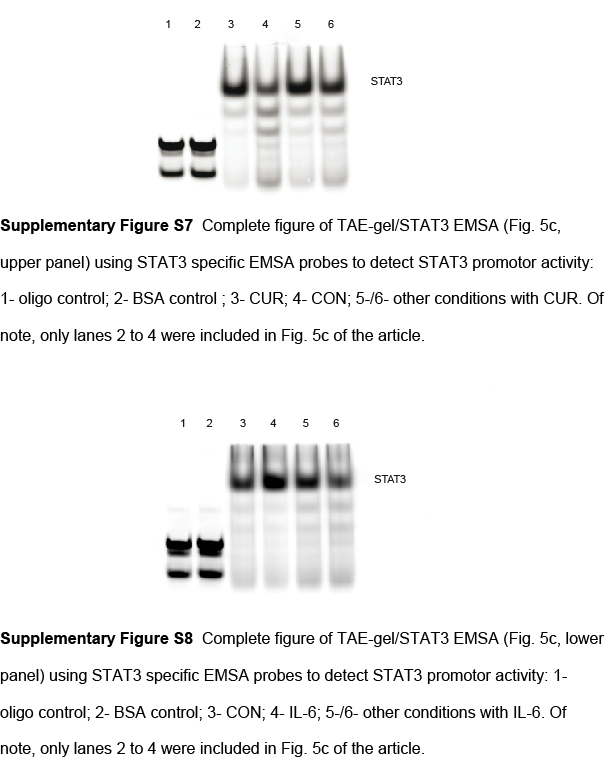


**
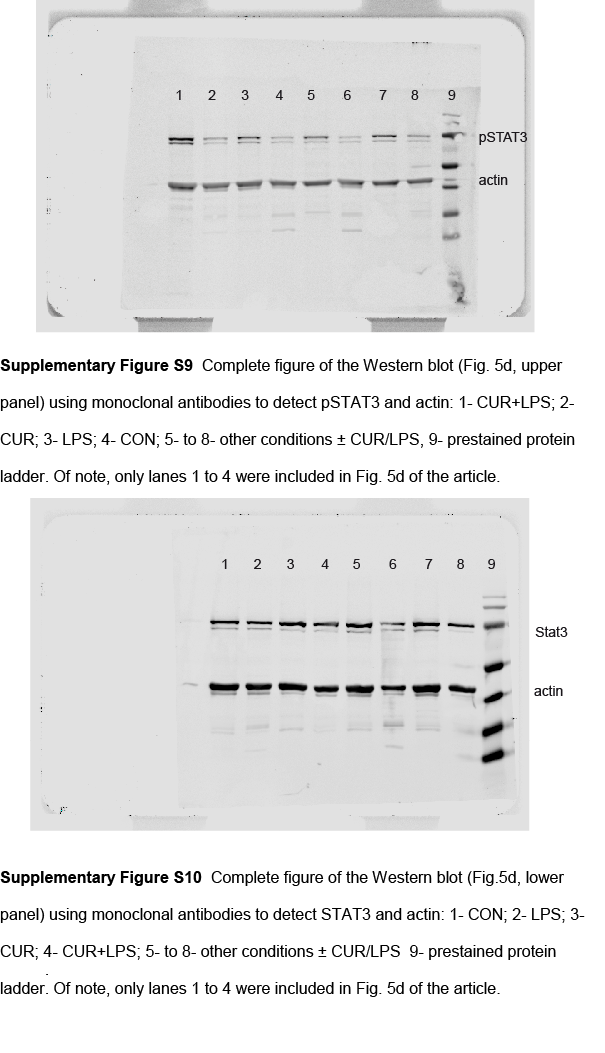
**


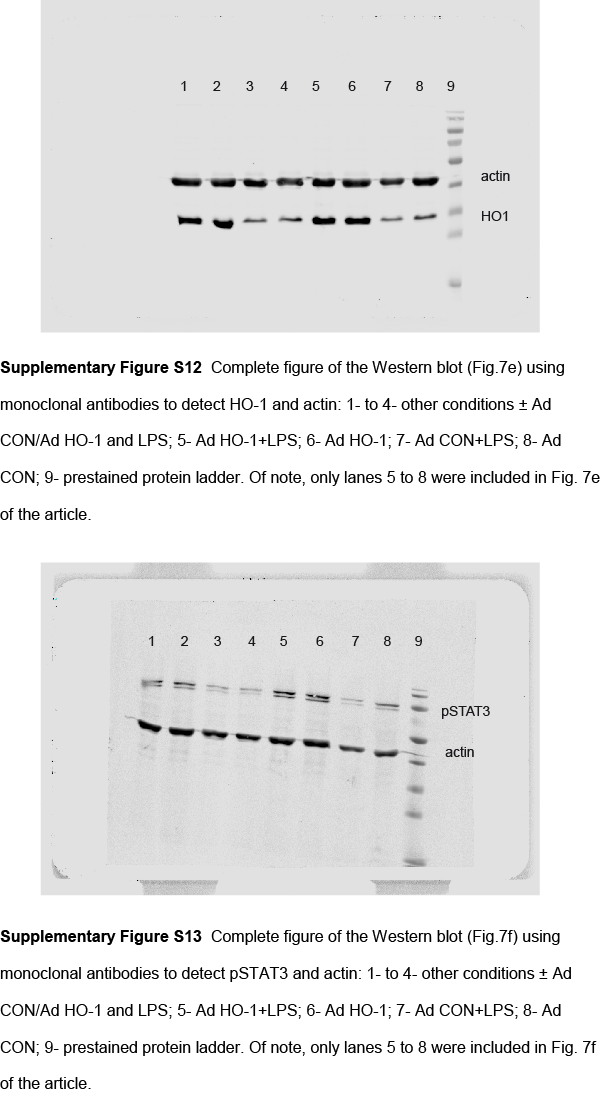

Supplement: Supplementary Information and Data [file srep44482-s1.doc]
